# TAPGFusion: Anatomy-Aware Triple-Attention and MRI-Conditioned Prior Learning for Multimodal Medical Image Fusion

**DOI:** 10.3390/bios16090458

**Published:** 2026-08-23

**Authors:** Liu Wang, Yang Zhou, Wenjia Li, Jian Zhao

**Affiliations:** 1 School of Computer Science and Technology, Changchun University, 6543 Weixing Road, Changchun 130022, China; wangl95@ccu.edu.cn (L.W.); 241501497@mails.ccu.edu.cn (Y.Z.); 241503553@mails.ccu.edu.cn (W.L.); 2 Jilin Provincial Key Laboratory of Human Health Status Identification Function & Enhancement, 6543 Weixing Road, Changchun 130022, China

**Keywords:** deep learning, detail-enhanced attention block, medical image fusion, multi-scale encoder, physiological prior-guided attention block

## Abstract

Multimodal medical image fusion combines anatomical and functional information from different imaging modalities. However, existing methods often struggle to preserve fine anatomical structures while incorporating complementary functional information. To address this problem, we propose TAPGFusion, an anatomy-aware multimodal medical image fusion network. It employs a Multi-Scale Encoder to capture fine local details and broad anatomical structures through parallel convolutions with different receptive fields. A Detail-Enhanced Attention Block further refines the extracted features through channel, spatial, and pixel attention. In addition, a Physiological Prior-Guided Attention Block dynamically balances anatomical and functional features using MRI-conditioned prior information and edge constraints. The main contribution of TAPGFusion is a unified framework that jointly addresses multi-scale feature representation, fine-grained feature selection, and spatially adaptive anatomical–functional fusion. Extensive experiments on three public medical imaging datasets demonstrate the effectiveness and robustness of the proposed method. TAPGFusion achieves CC values above 0.83 and SSIM values above 0.78 on the CT–MRI, PET–MRI, and SPECT–MRI fusion tasks. These results indicate that the proposed method effectively preserves anatomical structures while integrating complementary functional information.

## 1. Introduction

Single-modality imaging sensors typically capture only specific physical properties of human tissues, often failing to provide comprehensive, complementary pathological information [1,2]. For instance, computed tomography (CT) offers limited soft-tissue contrast and lacks physiological metabolic data [3]. Magnetic resonance imaging (MRI), while providing excellent structural detail, requires prolonged acquisition times and is insensitive to certain tissue structures and signal characteristics [4]. In contrast, positron emission tomography (PET) and single-photon emission computed tomography (SPECT) provide metabolic information. However, their relatively low spatial resolution and limited anatomical detail can reduce localization accuracy [5,6]. Consequently, the inherent expressive limitations of single-modality sensors impede a holistic characterization of the observed targets. Multimodal medical image fusion (MMIF) integrates complementary information from different imaging modalities. This improves the completeness and reliability of the fused images and supports subsequent quantitative analysis and clinical assessment [7].

Early feature-level fusion formed the core of MMIF methodologies, traditionally following a sequential pipeline: multi-sensor feature extraction, cross-sensor feature fusion, and image reconstruction. These approaches rely on fixed, handcrafted operators to extract features from the source images. As a result, they mainly capture low-level information, such as intensity, gradients, and basic structures, while failing to model deeper cross-modal relationships and semantic information [8]. Initial methods, predominantly represented by Principal Component Analysis (PCA) [9,10], achieved fusion by linearly transforming high-dimensional sensor features for dimensionality reduction and information aggregation. While computationally straightforward, PCA models only global linear relationships, risking the loss of critical local details like edges and textures and exhibiting high sensitivity to sensor noise and artifacts. To simultaneously preserve global structures and local details, wavelet transforms [11,12,13] were widely adopted, facilitating independent fusion of distinct frequency bands via multiresolution decomposition. Although wavelet methods alleviate some spatial limitations of PCA, they still depend on fixed basis functions and handcrafted filters. This limits their ability to represent directional structures and adapt to modality differences and complex anatomical patterns. To improve directional representation, multi-scale geometric methods such as the Non-Subsampled Contourlet Transform (NSCT) were introduced [14,15,16]. These methods better capture curves and edges, but they still rely on manually designed basis functions and lack data-driven adaptability. Subsequently, sparse representation [17,18] and low-rank representation [19,20,21] enabled feature fusion via data-driven dictionary learning, bolstering robustness against noise and redundant information, albeit at the cost of complex model optimization and low computational efficiency.

The advent of deep learning [22] has broken through the technical bottlenecks of early feature-level fusion. Early research primarily utilized Convolutional Neural Networks (CNNs) [23,24,25,26], leveraging their local receptive fields and weight-sharing properties to automatically extract deep features from raw multi-sensor signals and execute end-to-end fusion, effectively eliminating the reliance on handcrafted features. Nevertheless, CNNs are inherently limited by weak long-range dependency modeling and insufficient global context capture; when processing complex medical images with significant scale variations across sensors, CNNs frequently lose critical associative information between tissues and lesion regions [27,28]. To rectify this, Transformers have been introduced into the medical image fusion domain. By employing self-attention mechanisms to establish global multi-sensor contextual modeling and long-range dependencies, Transformers have significantly enhanced the representation of global structures and semantic information, leading to superior preservation of complementary multi-sensor observations [29,30,31,32].

Despite these advancements, existing multimodal medical image fusion methods still face several important limitations:

1. **Insufficient multi-scale representation:** A single convolutional receptive field cannot simultaneously capture fine local details and large-scale anatomical structures, which may lead to incomplete feature extraction.
2. **Inadequate feature discrimination:** Standard convolutions cannot explicitly distinguish the importance of features across channel, spatial, and pixel dimensions. As a result, redundant information and noise may be retained during fusion.
3. **Limited adaptability of fusion strategies:** Fixed or symmetric fusion weights cannot adapt to regional differences between anatomical and functional modalities. This may result in blurred anatomical boundaries or insufficient preservation of functional information.

These limitations motivate the design of TAPGFusion. The Multi-Scale Encoder addresses the first limitation by extracting features at different receptive-field scales. The DEA Block addresses the second limitation through channel, spatial, and pixel attention. The PGA Block addresses the third limitation by dynamically balancing anatomical and functional information using MRI-conditioned prior and edge information.

To resolve these issues, we propose TAPGFusion, a novel architecture tailored specifically for multimodal medical image fusion. We design a Multi-Scale Encoder featuring parallel branches: an upper branch employing dilated convolutions to extract global sensor features and a lower branch utilizing parallel 3 × 3 and 5 × 5 convolutions to preserve fine-grained details. Furthermore, we introduce a Detail-Enhanced Attention (DEA) Block that leverages channel attention to highlight critical tissue features, spatial attention to focus on anatomical structures, and pixel-wise attention to fine-tune individual pixel weights. Crucially, we construct a Physiological Prior-Guided Attention (PGA) Block that dynamically generates fusion weights driven by MRI-derived physiological priors and edge information, achieving optimal, context-aware multimodal integration.

The main contributions of this work are summarized as follows:

• We propose a Multi-Scale Encoder to overcome the inability of single-receptive-field convolutions to simultaneously capture fine details and global structures. By integrating multi-scale branches, this module achieves complementary feature extraction.
• We introduce a Detail-Enhanced Attention (DEA) Block to address the limitations of standard convolutions. Through a tripartite mechanism of channel, spatial, and pixel-wise attention, it precisely suppresses redundant noise and enhances targeted representation.
• We design a Physiological Prior-Guided Attention (PGA) Block to rectify the poor adaptability of fixed-weighting schemes. By dynamically generating fusion weights based on physiological priors, it intelligently preserves anatomical structures while emphasizing metabolically active zones.

The remainder of this paper is organized as follows. Section 2 reviews traditional and deep learning-based methods for multimodal medical image fusion. Section 3 presents the proposed TAPGFusion framework and describes its main components, including the Multi-Scale Encoder, the DEA Block, and the PGA Block. Section 4 introduces the experimental settings and reports the comparative, ablation, robustness, and visualization results. Finally, Section 5 concludes the paper and discusses future research directions.

## 2. Related Work

Multimodal medical image fusion methods can be broadly divided into traditional approaches and deep learning-based approaches. Traditional methods rely mainly on mathematical transformations, handcrafted feature representations, and predefined fusion rules. In contrast, deep learning-based methods automatically learn multimodal features and fusion strategies from data. This section reviews the development, representative methods, and main limitations of these two categories.

### 2.1. Traditional Methods

For decades, early feature-level fusion served as the cornerstone strategy for multimodal medical image fusion, primarily focusing on integrating data from multiple imaging sensors. Among the earliest techniques, Principal Component Analysis (PCA) was utilized to linearly aggregate raw sensor observation signals [9]. To mitigate the spectral distortion commonly induced by traditional PCA-based pan-sharpening, a block-based PCA transform was introduced, significantly enhancing adaptability to multi-sensor signals [10]. To address PCA’s inherent deficiencies in capturing spatial details, wavelet transforms were subsequently adopted, enabling the hierarchical processing of multi-resolution sensor data [11]. Several fusion strategies based on wavelet transforms were developed to preserve imaging details through the selective fusion of high- and low-frequency coefficients [12,13]. However, the limited directional descriptiveness of wavelet transforms rendered them inadequate for the complex structures inherent to multi-sensor imaging, prompting the introduction of the Non-Subsampled Contourlet Transform (NSCT). Methodologies employing NSCT facilitated multi-scale feature decomposition and reconstruction [14], utilized specific fusion rules on high- and low-frequency components [15], and processed sub-bands independently to improve detail retention [16]. Despite these improvements, NSCT still relied heavily on handcrafted rules and struggled to sufficiently suppress sensor noise and redundant information, a limitation that catalyzed the development of sparse representation methods. Techniques based on regional sparse representation were proposed to enhance the representation of blurred sensor imaging areas [18], alongside comprehensive frameworks extending sparse modeling from single to multiple components [17]. Ultimately, traditional methods are universally constrained by high computational overhead and weak adaptive capabilities, rendering them ill-suited for the complex characteristics of modern multi-sensor imaging and driving the paradigm shift toward advanced, data-driven methodologies.

### 2.2. Deep Learning-Based Methods

The rapid advancement of deep learning has effectively surmounted the limitations of early feature-level fusion in processing multi-sensor imaging information. Early architectures predominantly relied on Convolutional Neural Networks (CNNs), exploiting local receptive fields, weight sharing, and spatial invariance to directly learn features from raw sensor data. Pioneering CNN-based methods automated the extraction and fusion of multimodal sensor features [23], while deeper CNN designs were engineered to enhance the imaging quality of brain tumors by learning to quantify pixel activity and optimize fusion weights [24]. Furthermore, universal image fusion frameworks, such as IFCNN, utilized convolutional layers to extract salient features from multi-sensor inputs, adapting the fusion process prior to output reconstruction [26]. Nevertheless, CNNs are fundamentally handicapped by weak long-range dependency modeling and inadequate capture of global context, making it challenging to properly model extensive structural correlations across disparate sensors. To overcome these architectural constraints, Transformers were introduced to the fusion domain. Relying on self-attention mechanisms to achieve global sequence modeling [33], Transformer-based architectures like MM-Net have been developed to synergize anatomical and functional sensor imagery [29]. Additionally, unsupervised, multi-scale adaptive Transformer methods have been proposed to significantly bolster the network’s capacity to adaptively process the varied multi-scale characteristics of multi-sensor imaging [31]. Recent studies have further emphasized the importance of integrating complementary information from heterogeneous sensing modalities. Fan et al. [34] discussed multi-channel biomedical sensing for wound infection monitoring, while Zhou et al. [35] investigated synergistic high-order interactions for multimodal image fusion, highlighting the value of effective cross-modal information interaction.

## 3. Methods

### 3.1. Overall Architecture

**Figure 1:**
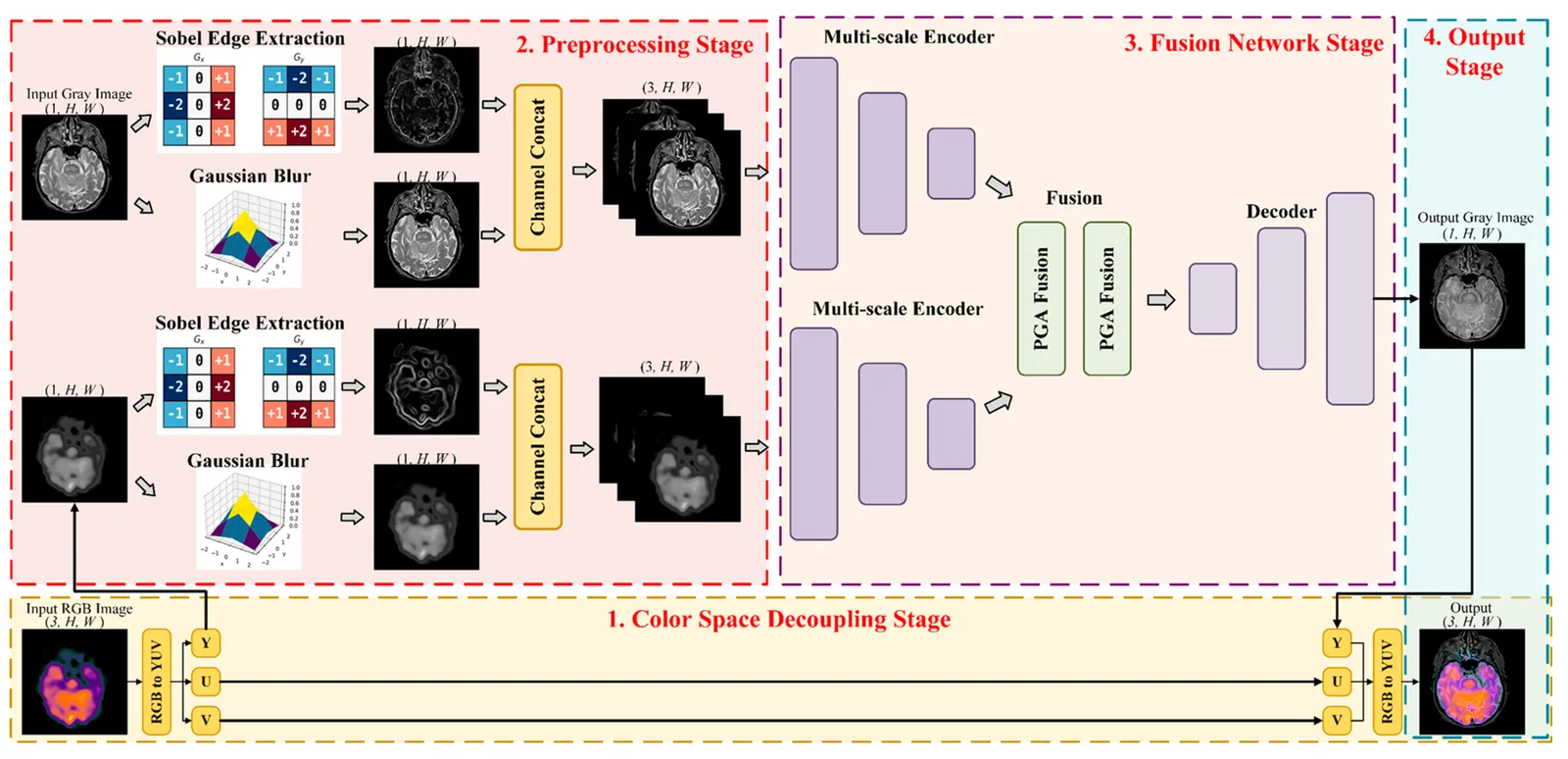
Overall architecture of the proposed TAPGFusion. The framework consists of four stages: color space decoupling, preprocessing, fusion network, and output. The luminance channel is extracted via RGB-to-YUV conversion, and preprocessing is performed using Gaussian blur and Sobel edge detection. Features from two different modalities are fed into two parallel Multi-Scale Encoders, then fused through the Gated Asymmetric Fusion Module (PGA Block). Finally, the fused image is reconstructed by the decoder.

The overall architecture of the proposed TAPGFusion is illustrated in Figure 1. Given two registered multimodal medical input images, $I_{A}\in R^{1\times H\times W}$ and $I_{B}\in R^{1\times H\times W}$, we propose an end-to-end deep learning framework that translates a dual-modality input into a single-modality output. The framework comprises four primary stages: input preprocessing, a Multi-Scale Encoder, physiological prior-guided fusion, and a decoder. The input images I_A_ and I_B_ undergo preprocessing via normalization, Gaussian blurring, and Sobel edge detection applied to the single-channel images. The normalized original image, smoothed features, and edge features are subsequently concatenated into a three-channel input, providing enriched textural and structural information for downstream feature extraction. Subsequently, the corresponding features of I_A_ and I_B_ are synchronously fed into the proposed Multi-Scale Encoder. This encoder employs a dual-branch topology to independently extract multi-scale details and global receptive field features. It embeds a Detail-Enhanced Attention Block (DEA Block)—which integrates channel, spatial, and pixel attention mechanisms—to fortify semantic representation and cross-modal alignment capabilities. In the fusion phase, the extracted dual-modality features are routed to the Physiological Prior-Guided Attention Block (PGA Block), a gated asymmetric fusion module. This block utilizes a lightweight edge detection head and a physiological prior network to generate dynamic gating weights, adaptively modulating the fusion ratio of the anatomical features from I_A_ and the functional features from I_B_ based on anatomical boundaries and functional distributions. This ensures the precise, weighted integration of anatomical structures and functional metabolic features. Finally, decoding and reconstruction are executed via two-stage transposed convolutional upsampling combined with residual convolutional blocks. The decoder progressively reconstructs the fused high-dimensional features into a single-channel image with the same spatial dimensions as the input. This design preserves anatomical structures and functional information while maintaining a lightweight architecture.

### 3.2. Multi-Scale Encoder

**Figure 2:**
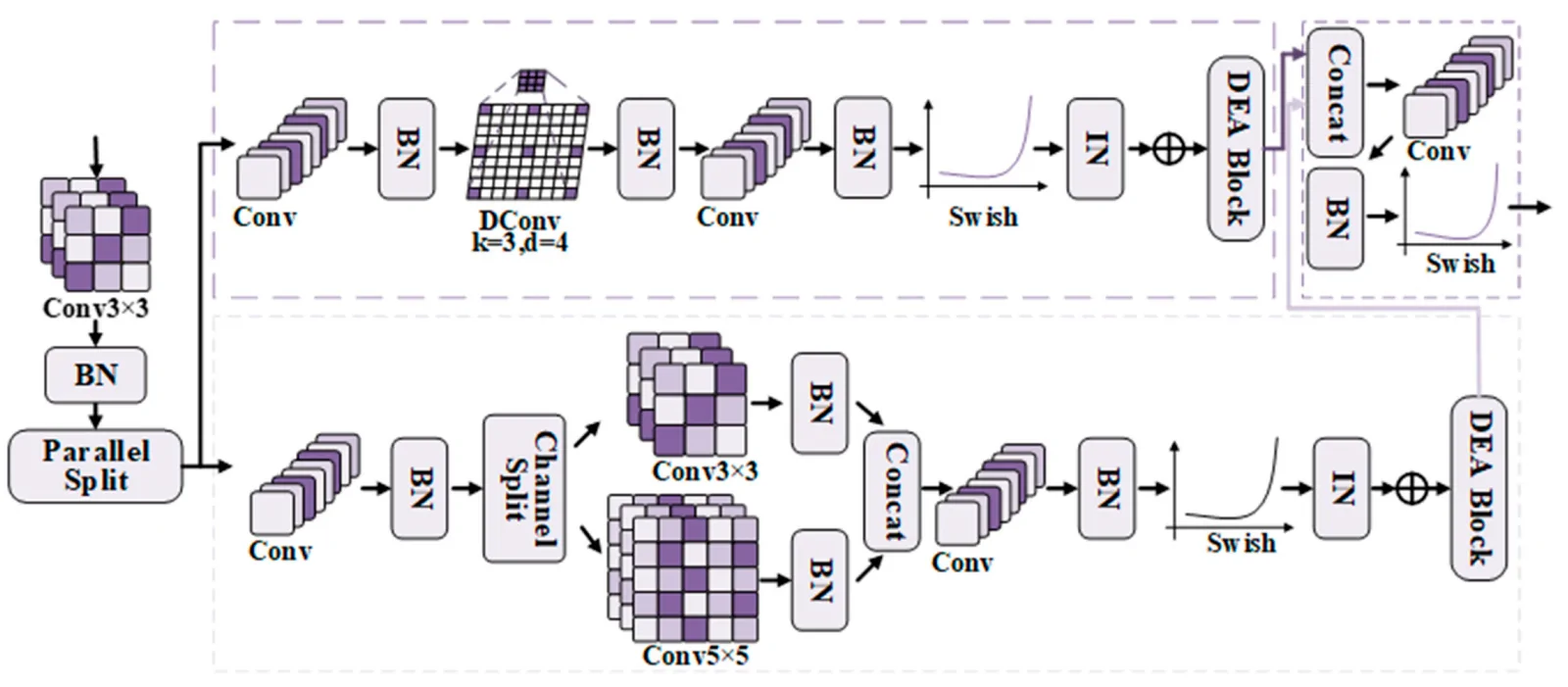
Structure of the Multi-Scale Encoder.

Conventional medical image fusion encoders often rely on a single receptive field. This limits their ability to capture fine local details and broad anatomical structures simultaneously. To address this issue, we designed the Multi-Scale Encoder shown in Figure 2. It extracts complementary multimodal features at different spatial scales. Initially, a cc convolution is applied to map the preprocessed input features to a unified dimensional space, expressed as:

(1) $$X_{0}=Conv_{3\times 3}\left(X\right)$$

where $X\in R^{C\times H\times W}$ represents the raw input feature to the multi-scale encoder, with C, H, and W denoting the number of channels, height, and width, respectively, and $X_{0}$ is the dimensionally mapped base feature.

To accurately capture pixel-level textures, edges, and fine granularities, the upper branch of the encoder utilizes small-receptive-field 3 × 3 convolutions to extract local details:

(2) $$P_{3}=ReLU\left(BN\left(Conv_{3\times 3}\left(X_{0}\right)\right)\right)$$

where $P_{3}$ denotes the detailed features extracted by the 3 × 3 convolution, $\mathrm{BN}\left(\cdot \right)$ indicates batch normalization utilized to accelerate convergence and stabilize the training process, and $\mathrm{ReLU}\left(\cdot \right)$the rectified linear unit activation function introducing non-linear feature expression.

Concurrently, the upper branch employs a parallel 5 × 5 convolution to expand the local receptive field, assisting in the capture of edge and texture correlations:

(3) $$P_{5}=ReLU\left(BN\left(Conv_{5\times 5}\left(X_{0}\right)\right)\right)$$

where $P_{5}$ is the output of the 5 × 5 parallel convolution. This parallel configuration enables multi-scale coverage of details within the upper branch.

At the terminus of the dual branches, the Detail-Enhanced Attention Block first generates primary residual features via two convolutional layers and a residual connection:

(4) $$R=Conv_{2}\left(ReLU\left(Conv_{1}\left(U\right)\right)\right)+U$$

where U is the input feature to the attention module, $Conv_{1}$ and $Conv_{2}$ are the convolutional operations within the module, and *R* is the feature map following preliminary convolution and residual fusion.

For the primary residual features, a channel attention module applies adaptive weighting along the channel dimension to filter critical modality features:

(5) $$M_{c}=\sigma \left(Conv_{1\times 1}\left(ReLU\left(Conv_{1\times 1}\left(AvgPool\left(R\right)\right)\right)\right)\right)$$

where $M_{c}$ is the channel attention mask, and $\mathrm{AvgPool}\left(\cdot \right)$ represents global average pooling, utilized to compress spatial dimensions and extract global channel information.

A spatial attention module focuses on critical anatomical regions while suppressing background noise:

(6) $$M_{s}=\sigma \left(Conv_{7\times 7}\left(S_{avg}⊕S_{max}\right)\right)$$

where $M_{s}$ is the spatial attention mask, $S_{avg}$ and $S_{max}$ are the outcomes of global average pooling and global max pooling along the channel dimension, respectively, and ⊕ denotes concatenation along the channel dimension.

The pixel attention module performs fine-grained pixel-level calibration to further strengthen detailed feature representation:

(7) $$M_{p}=\sigma \left(DWConv_{3\times 3}\left(M_{c}+M_{s}\right)\right)$$

where $M_{p}$ is the pixel attention mask, and $DWConv_{3\times 3}$ represents a 3 × 3 depthwise convolution.

To balance non-linear expression and gradient propagation stability, efficiently integrate detail and structural features, and maintain inference efficiency by controlling parameter volume, a lightweight strategy ultimately merges the dual-branch features to yield the final encoder output:

(8) $$F=Swish\left(BN\left(Conv_{1\times 1}\left(F_{s}⊕F_{m}\right)\right)\right)$$

where $F_{s}$ and $F_{m}$ are the output features from the upper and lower branches, respectively, and F is the final fused feature of the encoder.

### 3.3. Detail-Enhanced Attention Block (DEA Block)

**Figure 3:**
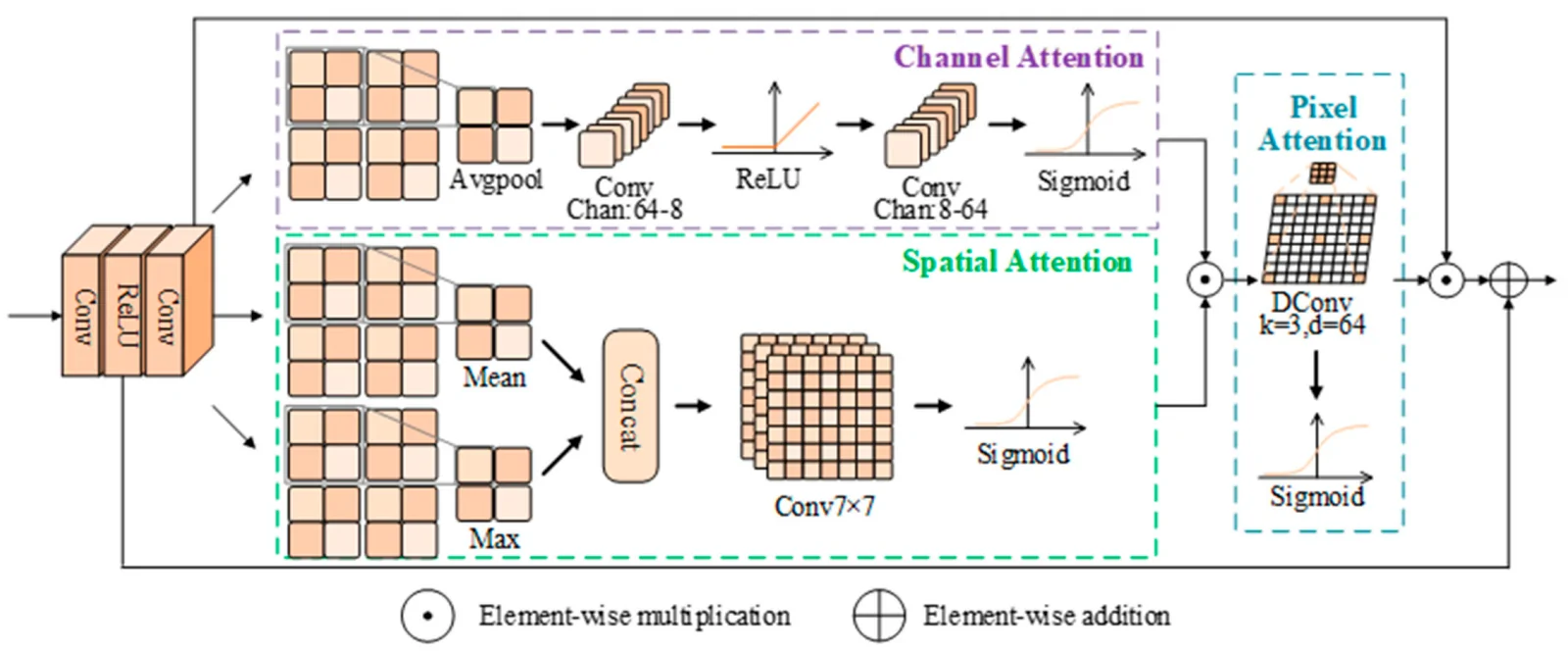
Architecture of the Detail-Enhanced Attention (DEA) Block.

To further enhance the discriminability and cross-modal complementarity of the features extracted by the Multi-Scale Encoder, as shown in Figure 3, we propose the Detail-Enhanced Attention Block (DEA Block) built upon the aforementioned multi-scale features. Let the input feature to the DEA Block be $U\in R^{C\times H\times W}$. A dual-layer convolution coupled with a primary residual connection is first employed to purify the base features, unearthing deep feature correlations while circumventing gradient vanishing and detail degradation:

(9) $$R=Conv_{2}\left(ReLU\left(Conv_{1}\left(U\right)\right)\right)+U$$

where $Conv_{1}$ and $Conv_{2}$ are the dual convolutional layers, $\mathrm{ReLU}\left(\cdot \right)$ is the non-linear activation function, and R is the preprocessed base feature serving the attention mechanism.

Following base purification, the module simultaneously activates the channel and spatial attention branches to perform adaptive feature filtering across different dimensions, purposefully eliminating redundant channels and attenuating background interference. The channel attention focuses on weighting high-value feature channels, while the spatial attention targets critical regions for weighting; together, they complementarily heighten feature specificity:

(10) $$M_{c}=\sigma \left(Conv_{1\times 1}\left(ReLU\left(Conv_{1\times 1}\left(AvgPool\left(R\right)\right)\right)\right)\right)$$

(11) $$M_{s}=\sigma \left(Conv_{7\times 7}\left(S_{avg}⊕S_{max}\right)\right)$$

where $M_{c}$ is the channel attention mask, $\mathrm{AvgPool}\left(\cdot \right)$ is global average pooling, and $\sigma \left(\cdot \right)$ is the Sigmoid activation function. $M_{s}$ is the spatial attention mask, $S_{avg}$ and $S_{max}$ are the global average and max pooling results across the feature channel dimension, respectively, and ⊕ denotes channel-wise concatenation.

For a more granular feature modulation, the module fuses the outputs of the previous two attention mechanisms and generates a pixel-level attention mask via depthwise convolution. This breaks the dimensional constraints of traditional attention, achieving independent pixel-wise weighting and calibration to emphasize edges and textural details:

(12) $$M_{p}=\sigma \left(DWConv_{3\times 3}\left(M_{c}+M_{s}\right)\right)$$

where $M_{p}$ is the pixel attention mask and $DWConv_{3\times 3}$ is a 3 × 3 depthwise convolution.

Finally, the pixel attention mask is element-wise multiplied with the base feature and combined with a secondary residual connection to produce the final enhanced feature. This highlights valid information via attention while stabilizing feature propagation through the residual structure to avert feature distortion:

(13) $$O=R⊗M_{p}+U$$

where ⊗ denotes element-wise multiplication, and O is the module’s output feature, with the dual residual architecture solidifying the feature propagation pathway.

### 3.4. Physiological Prior-Guided Attention Block (PGA Block)

**Figure 4:**
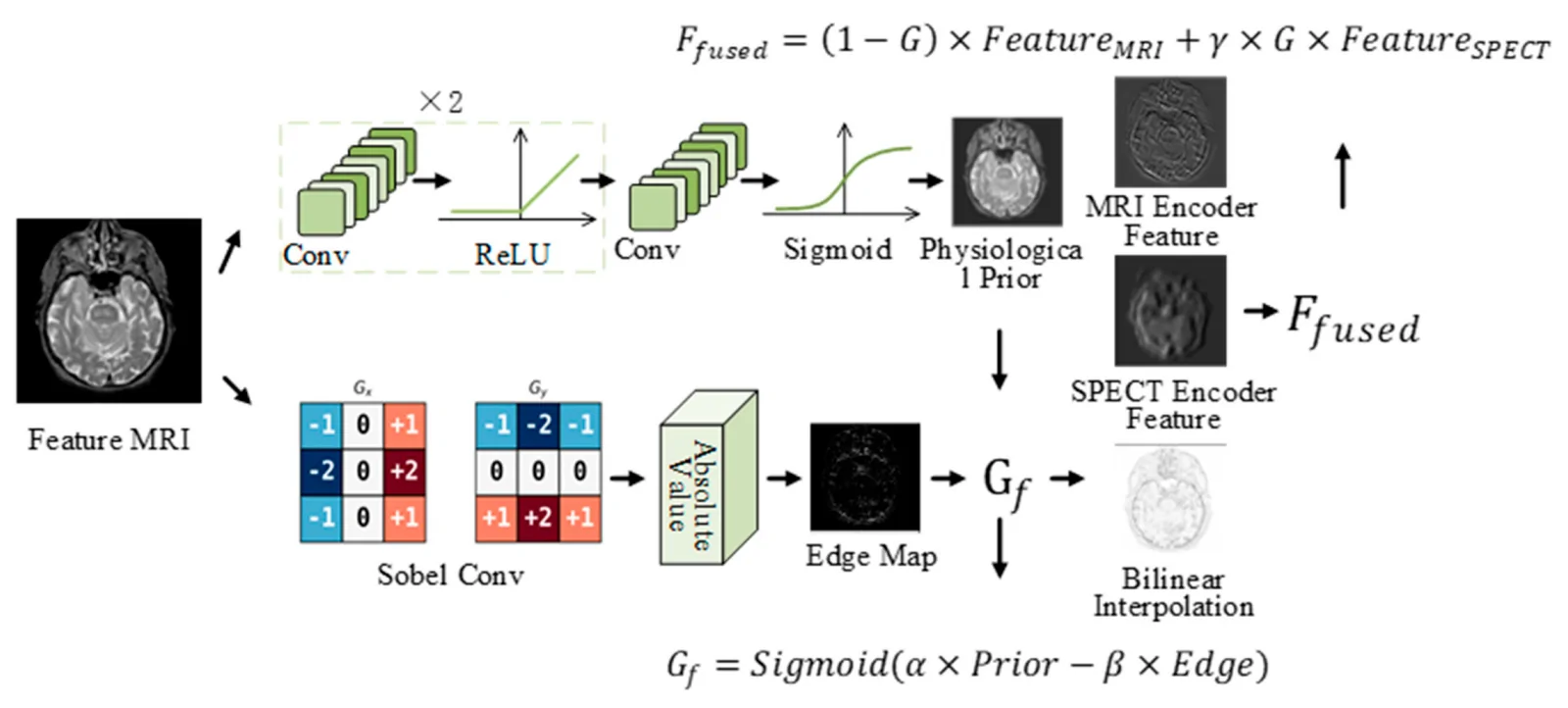
Architecture of the Physiological Prior-Guided Attention (PGA) Block.

Existing multimodal medical image fusion methods often rely on fixed fusion weights that fail to accommodate the varying feature requirements of different physiological regions, typically resulting in blurred anatomical structures or eclipsed functional information. As shown in Figure 4, to resolve this, we propose the Physiological Prior-Guided Attention Block (PGA Block).

This module takes the dual-modality features outputted by the encoder, $F_{MRI},F_{SPECT}\in R^{C\times H’\times W’}$, and the original anatomical image $I_{MRI}\in R^{1\times H\times W}$ as inputs. It constructs a dynamic gating mechanism through edge detection and physiological prior mining to adaptively adjust the fusion weights among different modality features. First, a learnable edge-detection operator extracts tissue boundaries and structural contours from the anatomical image. The resulting edge map is then resized to match the spatial resolution of the encoder features.

(14) $$E_{↑}=U\left(Conv_{Sobel}\left(I_{MRI}\right)\right)$$

where $Conv_{Sobel}$ is a 3 × 3 convolutional layer initialized with a fixed Sobel operator, and $U\left(\cdot \right)$ denotes bilinear interpolation upsampling to $R^{1\times H’\times W’}$ to match the spatial resolution of the encoded output features.

To unearth the implicit physiological tissue distribution priors and strictly align the spatial dimensions of the prior map with the features $F_{MRI}$ and $F_{SPECT}$ for semantic-level guidance, a lightweight convolutional network regresses the physiological prior map from the raw anatomical image and upsamples it: Here, the term “physiological prior” refers to an MRI-conditioned latent spatial weighting map rather than an externally provided physiological annotation or manually predefined region-of-interest mask. The prior-generation branch is jointly optimized with the overall fusion network and does not receive direct supervision from lesion labels, segmentation masks, or physiological-region annotations. Therefore, the MRI input and edge response provide an explicit anatomy-related inductive bias, while the specific spatial distribution of the prior map is learned end-to-end from the fusion objective.

(15) $$P_{↑}=U\left(\sigma \left(Conv_{1\times 1}\left(ReLU\left(Conv_{3\times 3}\left(ReLU\left(Conv_{3\times 3}\left(I_{MRI}\right)\right)\right)\right)\right)\right)\right)$$

where $\sigma \left(\cdot \right)$ is the Sigmoid activation function, and $P_{↑}\in {\left[0,1\right]}^{1\times H’\times W’}$ is the normalized physiological prior map.

The module adaptively balances anatomical and functional information. It assigns greater weight to the functional modality in regions with strong physiological priors and preserves anatomical details where edge responses are prominent. This balance is controlled by a gating weight derived from the physiological prior and edge information.

(16) $$G_{f}=\sigma \left(\alpha \cdot P_{↑}-\beta \cdot E_{↑}\right)$$

where $\alpha ,\beta \in R^{+}$ are learnable parameters, and $G\in {\left[0,1\right]}^{1\times H’\times W’}$ is the fusion gating weight, maintaining spatial resolution completely identical to $F_{MRI}$ and $F_{SPECT}$.

Ultimately, to execute dynamic fusion within the feature space, ensuring spatial complementarity between anatomical and functional features while mitigating fusion biases caused by inter-modal numerical distribution disparities, the module applies pixel-wise adaptive fusion to the dual-modality features using the gating weights. This generates a fused feature that possesses both structural integrity and functional discriminability:

(17) $$F_{fused}=\left(1-G\right)⊙F_{MRI}+\gamma \cdot G⊙F_{SPECT}$$

where $\gamma \in R^{+}$ is a learnable scaling factor, ⊙ denotes element-wise multiplication, and $F_{fused}\in R^{C\times H’\times W’}$ represents the fused feature to be subsequently fed into the decoder.

The proposed architecture is designed according to three complementary considerations. First, multimodal medical images contain both fine tissue boundaries and large-scale anatomical structures. Therefore, parallel convolutional branches with different receptive fields are adopted to capture information at multiple spatial scales. Second, features from different modalities exhibit varying importance across channels, spatial regions, and individual pixels. This motivates the use of channel, spatial, and pixel attention in the DEA Block. Third, MRI generally provides clearer anatomical structures, whereas PET and SPECT mainly convey functional activity. Accordingly, the PGA Block adopts an asymmetric fusion strategy that uses MRI-conditioned prior information and edge constraints to regulate the incorporation of functional features. This design preserves anatomical structures while adaptively emphasizing functional information in relevant regions, without requiring additional anatomical annotations or physiological labels.

## 4. Experiments

This section evaluates the proposed TAPGFusion framework from multiple perspectives. First, the datasets, implementation details, and evaluation metrics are introduced. The proposed method is then compared with representative state-of-the-art approaches on the CT–MRI, PET–MRI, and SPECT–MRI fusion tasks. Statistical significance and ablation analyses are further conducted to assess the reliability of the improvements and the contributions of the individual modules. Finally, noise, modality degradation, and intermediate-feature visualization experiments are presented to evaluate the robustness and feature representation capability of the proposed method.

### 4.1. Datasets

To evaluate the performance of the proposed TAPGFusion method, we used the publicly available Harvard Medical Image Database for three representative multimodal medical image fusion tasks: CT–MRI, PET–MRI, and SPECT–MRI. Following the dataset selection and partitioning protocol adopted in [36], we selected 184 registered CT–MRI image pairs, 269 registered PET–MRI image pairs, and 357 registered SPECT–MRI image pairs. For each modality combination, 24 image pairs were randomly selected as an independent test set, while the remaining image pairs were used for model training. Consequently, the training sets consisted of 160 CT–MRI pairs, 245 PET–MRI pairs, and 333 SPECT–MRI pairs, respectively.

All source images had a spatial resolution of 256×256 pixels and were directly used as the network inputs without cropping or resizing. Before being fed into the network, the pixel intensities of each source image were normalized to the range of $\left[01\right]$. The same preprocessing procedure was consistently applied to the training and test sets.

All images had a spatial resolution of 256 × 256 pixels. CT and MRI images were single-channel grayscale images, whereas PET and SPECT images were color images. For the PET–MRI and SPECT–MRI fusion tasks, the functional images were converted into the YCbCr color space. The Y channel, which contains the primary luminance and structural information, was used as the input to the fusion network together with the corresponding MRI image. After fusion, the resulting Y channel was recombined with the original Cb and Cr channels to reconstruct the final color fused image.

The dataset partitioning was performed at the level of registered image pairs before model training, and the same partition was used throughout all experiments. No image pair was included in both the training and test sets, thereby preventing direct overlap between the two subsets. The test sets were kept separate from the training process and were used only for the final quantitative and qualitative evaluation.

### 4.2. Implementation Details

All experiments in this study were implemented using the PyTorch 2.1.2 framework and executed on an NVIDIA (Santa Clara, CA, USA) GeForce RTX 4090 (24 GB) GPU. Tailored preprocessing strategies were devised to accommodate the distinct imaging characteristics and data profiles of different medical modalities. For the CT-MRI fusion task, since both CT and MRI source images are grayscale, they were directly fed into the model without any color space conversion. Conversely, for the PET-MRI and SPECT-MRI fusion tasks, where PET and SPECT are RGB images, we adopted the color space conversion strategy proposed in [37], uniformly mapping the SPECT/PET images into the YUV color space. Following conversion, only the Y channel—which encapsulates the core structural and luminance features of the image—was extracted as the training input for the fusion model. The Cb and Cr channels, which carry chrominance information but lack practical medical diagnostic value, were strictly aligned spatially with the corresponding Y channel, temporarily stored, and excluded from the model training process. Once the cross-modal fusion computations between the PET-Y/SPECT-Y channels and the MRI images were completed to generate the fused Y channel, the previously stored original Cb and Cr channels from the PET/SPECT images were recombined with the fused Y channel. An inverse YUV-to-RGB transformation was then applied to reconstruct and output the final PET-MRI and SPECT-MRI fused images, seamlessly integrating the fused structural information with the original color representation. This strategy successfully decouples structural and color information, maintaining the visual interpretability of the fused images while ensuring the efficacy of cross-modal fusion feature learning.

Training Strategy: All models were trained using the AdamW optimizer with a learning rate set to 1 × 10^−3^. The training process spanned 100 epochs with a batch size of 4.

Loss Function: We adopted the loss function formulated in [36], which comprises three components: content loss, gradient loss, and structural similarity loss. Integrating these three distinct losses circumvents the limitations inherent to utilizing a singular loss function.

• Content Loss constrains the pixel-intensity consistency between the fused image and the source images, guaranteeing the retention of foundational grayscale and luminance information from each modality.
• Gradient Loss focuses on high-frequency details such as image edges and textures; by constraining gradient information, it ensures the fused image possesses clear structures and sharp edges, preventing detail blurring.
• Structural Similarity Loss evaluates image similarity across three dimensions—luminance, contrast, and structural information—ensuring the fused result remains consistent with the source images in terms of overall anatomical structure and visual perception.

To balance the three optimization objectives, the weights of the content loss, gradient loss, and structural similarity loss were set to 0.5, 0.5, and 1.0, respectively. The larger weight assigned to the structural similarity loss emphasizes the preservation of anatomical structures, while the content and gradient losses jointly constrain intensity information and fine details. These weights were kept fixed across all datasets and experiments.

**Computational Cost:** We further evaluated the computational complexity of TAPGFusion using one 256×256 multimodal image pair with a batch size of 1. The complete model contains approximately 1.59 million trainable parameters and requires 14.44 GFLOPs for a single forward pass. The multi-scale encoding stage constitutes the main computational component, accounting for approximately 0.84 million parameters and 8.76 GFLOPs because of its parallel convolutional branches. The DEA Blocks require approximately 0.28 million parameters and 2.38 GFLOPs, whereas the lightweight PGA Block introduces only approximately 0.05 million parameters and 0.34 GFLOPs. The decoder accounts for approximately 0.42 million parameters and 2.86 GFLOPs. Overall, the DEA and PGA Blocks introduce only moderate additional computation, indicating that the improved fusion performance is mainly attributable to the proposed feature-enhancement and adaptive-fusion mechanisms rather than a substantial increase in model size.

### 4.3. Evaluation Metrics

To quantitatively evaluate fusion performance, we employed seven metrics: Correla tion Coefficient (CC), Entropy (EN), Feature Mutual Information (FMI), Mutual Information (MI), Mean Squared Error (MSE), Peak Signal-to-Noise Ratio (PSNR), and Structural Similarity Index (SSIM).

### 4.4. Comparison with State-of-the-Art Methods

To benchmark the proposed TAPGFusion framework, we selected six current main stream state-of-the-art fusion methods for comparison: CDDFuse [38], FATFusion [39], GeSeNet [25], PMGI [40], SwinFuse [41], U2Fusion [42] and IFCNN [34].

#### 4.4.1. CT-MRI Dataset

The quantitative evaluation results on the CT–MRI dataset are presented in Table 1. The proposed method achieves the best performance in four key metrics, including CC, MSE, PSNR, and SSIM, demonstrating a favorable balance between structural preservation and reconstruction fidelity. Specifically, TAPGFusion obtains the highest CC of 0.8333, exceeding the second-best SwinFuse result of 0.8313. This improvement indicates that the fused images generated by our method maintain stronger overall correlation and content consistency with the source modalities.

TAPGFusion also achieves the lowest MSE of 0.0265 and the highest PSNR of 63.9868. Compared with the second-best PMGI results of 0.0267 in MSE and 63.9416 in PSNR, these values indicate that the proposed method introduces less reconstruction distortion while preserving source-image information more accurately. In addition, our method obtains the highest SSIM of 0.7884, slightly exceeding U2Fusion at 0.7877 and clearly outperforming the remaining comparison methods. The leading SSIM result further demonstrates the ability of TAPGFusion to preserve structural relationships, tissue boundaries, and local contrast during the fusion process.

Although FATFusion achieves the highest EN of 5.9146, PMGI obtains the highest MI of 2.7338, and U2Fusion achieves the highest FMI of 0.6599, these individual advantages are not consistently maintained across the other evaluation metrics. In contrast, TAPGFusion achieves the best results in all four reconstruction- and structure-related metrics while maintaining competitive information-preservation performance. This result suggests that the proposed method does not simply maximize image entropy or feature information independently. Instead, it seeks a more balanced integration of complementary CT and MRI information while controlling structural distortion.

**Figure 5:**
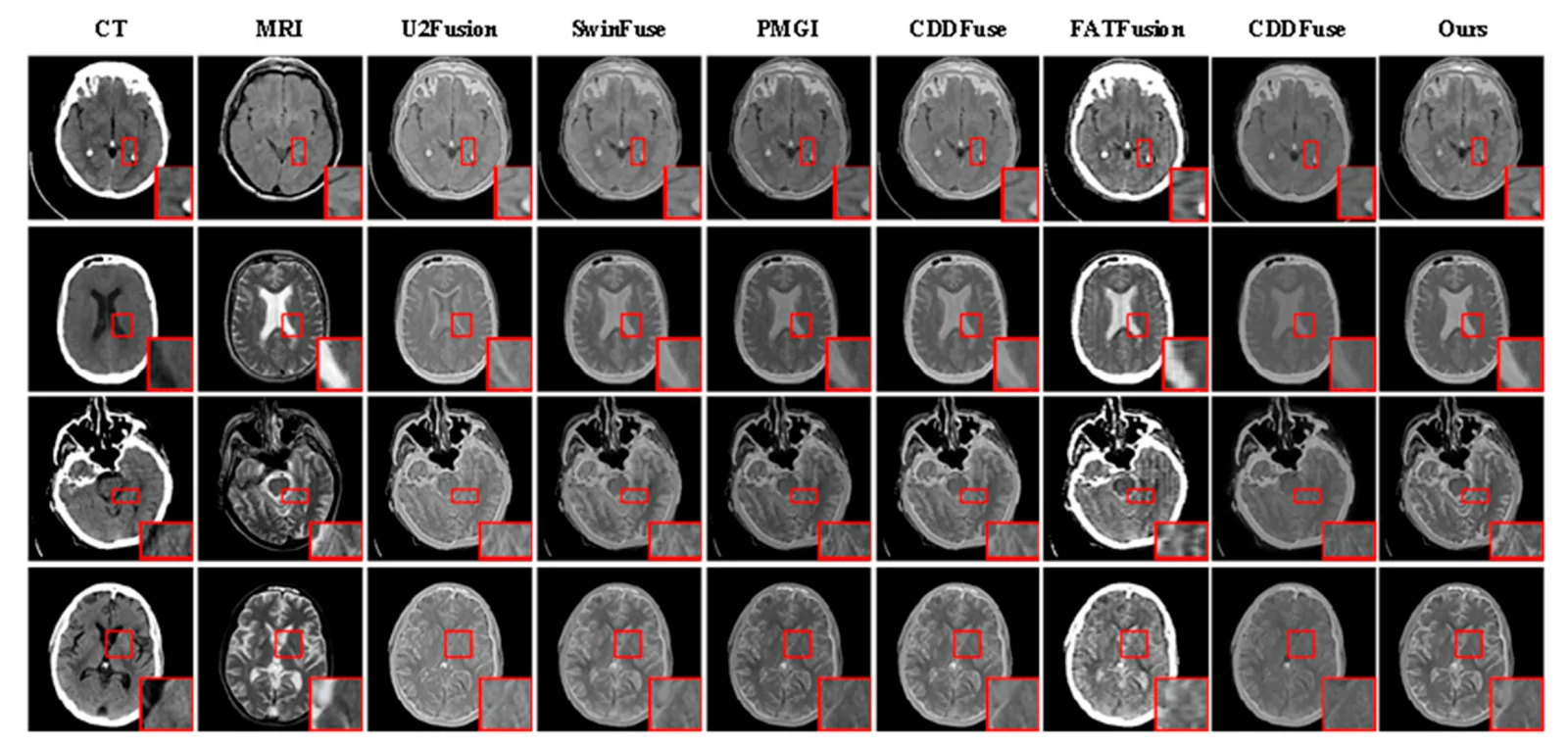
Qualitative comparison between the proposed method and six competing approaches on the CT-MRI dataset. Each row represents a different sample, and each column represents a different method.The red boxes denote the zoomed-in regions.

The visual comparisons in Figure 5 further support the quantitative findings. The fused images generated by our method preserve both the high-contrast skeletal structures of CT and the soft-tissue details of MRI. In comparison, U2Fusion and SwinFuse exhibit localized blurring or insufficient contrast in some regions, whereas CDDFuse and FATFusion show varying degrees of over-enhancement or luminance imbalance. The proposed method provides clearer structural boundaries, more natural intensity transitions, and fewer visible artifacts. These observations are consistent with its leading CC, MSE, PSNR, and SSIM results and demonstrate its effectiveness in balancing structural detail preservation and multimodal information integration.

#### 4.4.2. PET-MRI Dataset

The quantitative assessment results on the PET–MRI dataset are presented in Table 2. TAPGFusion achieves the best performance in six of the seven evaluation metrics, including CC, EN, MI, MSE, PSNR, and SSIM. This consistent performance demonstrates the effectiveness of the proposed method in preserving anatomical structures, retaining complementary functional information, and reducing fusion distortion.

Specifically, the CC of TAPGFusion reaches 0.8691, exceeding the second-best GeSeNet result of 0.8679. The highest CC indicates stronger overall consistency between the fused image and the PET and MRI source images. The proposed method also achieves the highest EN of 5.1583, compared with the second-best value of 5.1181 obtained by GeSeNet. This result suggests that TAPGFusion retains a relatively rich amount of information in the fused output without relying on excessive contrast enhancement.

In terms of complementary information preservation, TAPGFusion achieves the highest MI of 3.4233, surpassing the second-best SwinFuse result of 3.3808. This improvement indicates that the fused images contain more shared information from the anatomical and functional modalities. Moreover, TAPGFusion obtains the lowest MSE of 0.0280 and the highest PSNR of 63.7154, compared with the corresponding second-best GeSeNet results of 0.0281 and 63.7028. These results demonstrate that the proposed method reduces reconstruction errors while maintaining high signal fidelity.

TAPGFusion also achieves the highest SSIM of 0.7886, outperforming the second-best FATFusion result of 0.7817. The improvement in SSIM confirms that the proposed method effectively preserves the anatomical structures and local spatial relationships provided by MRI. Although PMGI achieves the highest FMI of 0.6442, its performance in the remaining metrics is substantially lower, particularly in CC, EN, MSE, PSNR, and SSIM. Therefore, the FMI result should be interpreted together with the other metrics. Overall, TAPGFusion provides a more balanced fusion result by jointly preserving MRI structural information and PET metabolic information.

**Figure 6:**
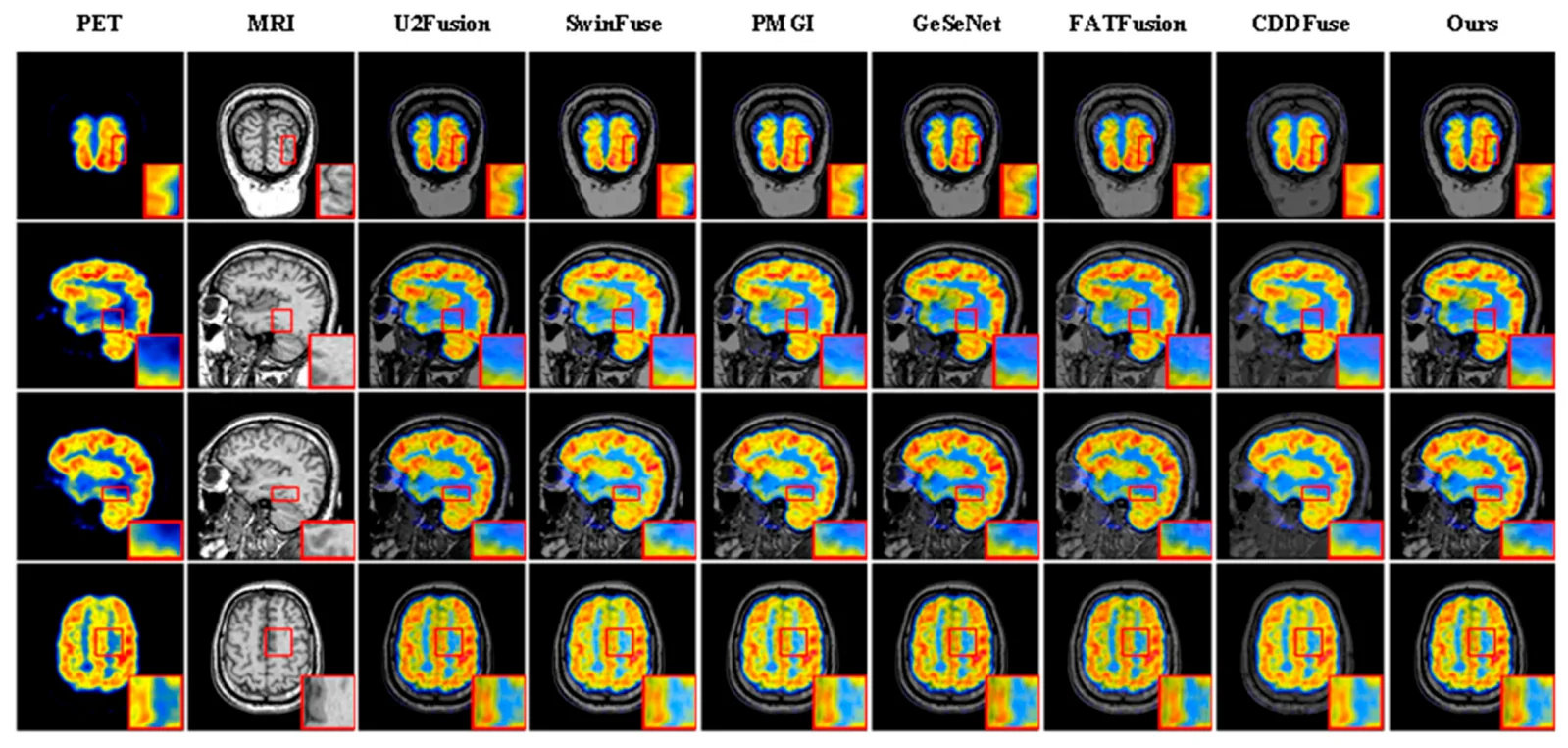
Qualitative comparison between the proposed method and six competing approaches on the PET-MRI dataset. Each row represents a different sample, and each column represents a different method. The red boxes denote the zoomed-in regions.

The visual comparisons in Figure 6 further support these quantitative findings. Our method preserves the metabolic information from PET while presenting the anatomical structures from MRI more clearly. The fused images exhibit sharper tissue boundaries, more natural color transitions, and fewer visible artifacts. In contrast, some comparison methods, such as CDDFuse and PMGI, show varying degrees of detail loss, structural blurring, or luminance distortion. The agreement between the quantitative and qualitative results confirms the overall effectiveness of TAPGFusion for PET–MRI image fusion.

#### 4.4.3. SPECT-MRI Dataset

**Figure 7:**
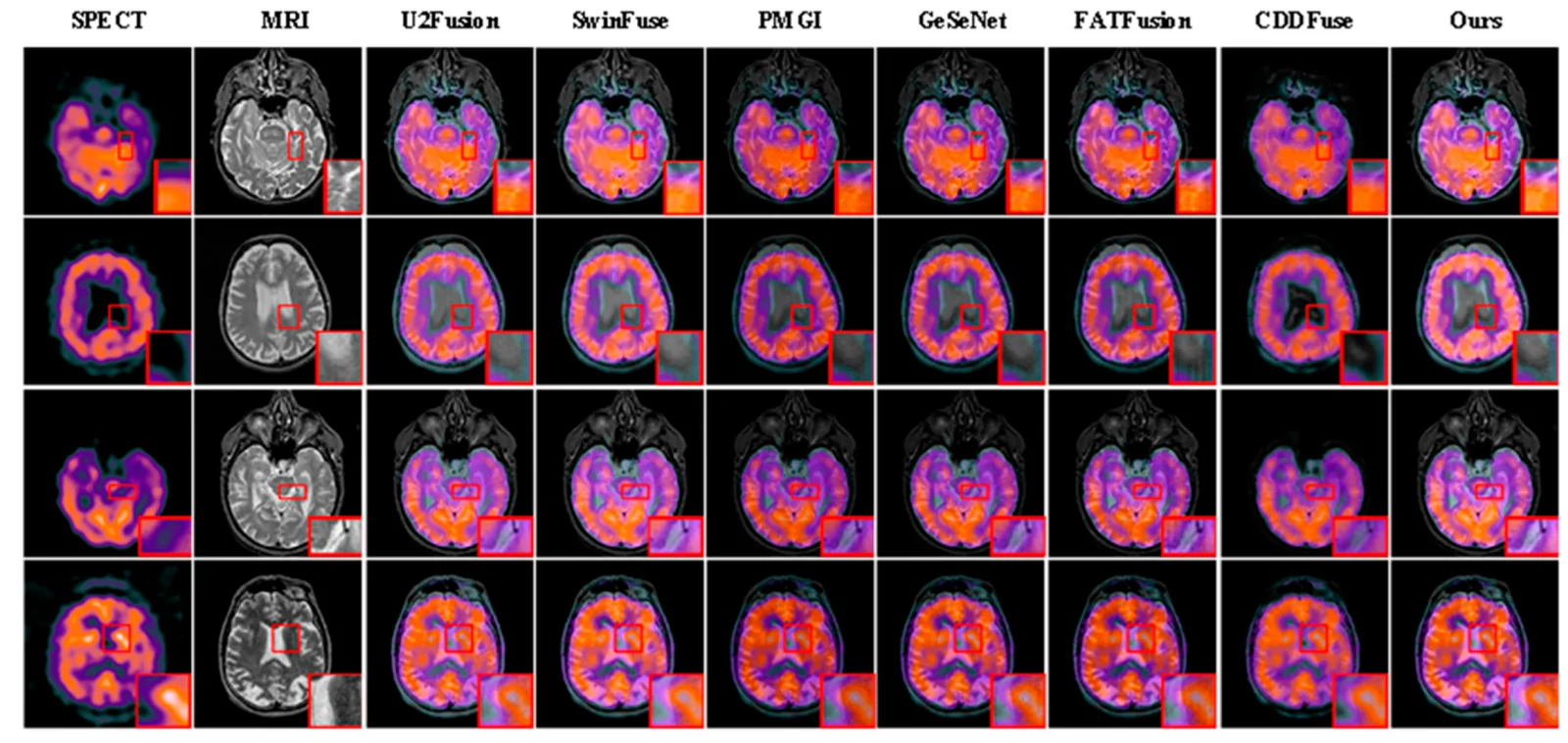
Qualitative comparison between the proposed method and six competing approaches on the SPECT-MRI dataset. Each row represents a different sample, and each column represents a different method. The red box indicates local zoomed-in details.

On the SPECT-MRI dataset, the quantitative results in Table 3 strongly corroborate the efficacy of our three core innovations. First, the superior performance in CC (0.9073), MI (2.8128), and SSIM (0.8692) validates the *Multi-Scale Encoder*: by parallelizing dilated convolutions (global context) with multi-kernel branches (local details), the module ensures comprehensive feature extraction that preserves both macro-anatomical consistency and fine-grained structural correlation across modalities. Second, the highest EN (4.2259)—reflecting maximal information density and high-frequency detail retention—directly evidences the contribution of the *Detail-Enhanced Attention (DEA) Block*: its tripartite channel-spatial-pixel attention mechanism adaptively amplifies diagnostically critical features (e.g., lesion boundaries, metabolic edges) while suppressing modality-specific noise, thereby enriching texture fidelity without over-smoothing. The joint improvements in structural metrics, such as SSIM and PSNR, and information-based metrics, such as MI and EN, demonstrate the effectiveness of the PGA Block. The block dynamically adjusts the fusion weights using MRI-conditioned prior information and edge constraints. It preserves anatomical structures in regions with strong edge responses while emphasizing functional information in metabolically active regions. The slight trade-offs in FMI and MSE indicate that the method does not optimize every metric independently. Instead, it aims to achieve a balanced preservation of structural and functional information. This interpretation is also supported by the visual results, which show natural color transitions, clear edges, and accurate spatial correspondence between anatomical and functional features. Collectively, these quantitative–qualitative alignments substantiate that TAPGFusion’s architecture—multi-scale extraction, triple-attention enhancement, and prior-guided adaptive fusion—synergistically resolves the core challenge of simultaneous anatomical integrity and functional validity in multimodal medical image fusion. As shown in Figure 7.

#### 4.4.4. Statistical Significance Analysis

To further evaluate the reliability of the performance improvements, paired-sample t-tests were conducted between the proposed TAPGFusion method and each comparison method based on the per-image metric values obtained from the same test image pairs. The significance level was set to p<0.05.

On the CT–MRI dataset, statistically significant differences were observed in CC, MSE, PSNR, and SSIM. On the PET–MRI dataset, the differences in MI, MSE, PSNR, and SSIM were statistically significant. On the SPECT–MRI dataset, statistically significant differences were obtained in CC, EN, MI, MSE, PSNR, and SSIM. In all these comparisons, the corresponding p-values were lower than 0.05.

These results demonstrate that the performance improvements achieved by TAPGFusion on the key evaluation metrics are statistically reliable rather than being caused by random variations among the test samples. Moreover, the significant differences in both reconstruction-related metrics, including MSE, PSNR, and SSIM, and information-preservation metrics, including CC, MI, and EN, further confirm the consistent ability of the proposed method to preserve anatomical structures and complementary functional information.

### 4.5. Ablation Study

To clarify the experimental workflow, all ablation configurations were predefined before training and evaluated as independent controlled experiments. They were not obtained through sequential backtracking or by progressively modifying a previously trained model according to the observed results. For each configuration, the network was independently initialized and trained using the same dataset partition, preprocessing procedure, optimizer, learning rate, batch size, number of epochs, and loss settings. Only the module or component under investigation was changed, while all other experimental conditions remained fixed.

The ablation analysis was conducted at two levels. First, module-level experiments were performed on the CT–MRI, PET–MRI, and SPECT–MRI datasets to evaluate the contributions of the Multi-Scale Encoder, DEA Block, and PGA Block. Second, component-level experiments were conducted on the PET–MRI dataset to examine the channel, spatial, and pixel attention branches within the DEA Block, as well as the MRI-conditioned prior, edge guidance, and adaptive gating mechanism within the PGA Block. Each trained variant was evaluated on the same independent test set, and the average CC, EN, FMI, MI, MSE, PSNR, and SSIM values were calculated for comparison.

The expanded ablation study in Table 4, Table 5 and Table 6 includes the baseline network and all possible combinations of the Multi-Scale Encoder, DEA Block, and PGA Block. The single-module configurations provide a direct assessment of the independent contribution of each component, whereas the two-module and full configurations reveal their interactions.

Compared with the baseline network, introducing each module individually improves several evaluation metrics, although the magnitude of improvement varies across modalities. The Multi-Scale Encoder generally improves multi-scale feature representation and structural correlation. The DEA Block enhances feature selection and local detail representation, while the PGA Block improves the adaptive balance between anatomical and functional information.

The results also indicate that the effects of the three modules are not simply additive. Certain partial combinations achieve better values for individual metrics but may reduce performance on other criteria. In contrast, the complete TAPGFusion model achieves the best or near-best results across the majority of metrics on all three datasets, demonstrating more balanced fusion performance.

Specifically, on the CT–MRI dataset, the complete model achieves the highest CC, EN, MI, and SSIM and the lowest MSE. On the PET–MRI dataset, it obtains the highest CC, FMI, MI, and SSIM while maintaining competitive reconstruction performance. On the SPECT–MRI dataset, it achieves the highest CC, EN, FMI, MI, PSNR, and SSIM. These results confirm both the independent effectiveness and complementary interaction of the three proposed modules.

#### 4.5.1. Component-Wise Ablation of the DEA Block

To further investigate the contribution of the internal components of the DEA Block, we conducted component-wise ablation experiments on the PET–MRI dataset. The PET–MRI task was selected because it simultaneously involves high-resolution anatomical information from MRI and functional metabolic information from PET, providing a representative setting for evaluating the attention mechanisms.

We separately removed the channel, spatial, and pixel attention branches while retaining the remaining network structure. When an attention branch was removed, its corresponding attention mask was replaced with an identity mask, while the residual convolutional path remained unchanged. All variants were trained using the same dataset partition, optimization settings, loss functions, and training epochs.

As shown in Table 7, the complete DEA Block achieves the best overall performance, demonstrating the complementary contributions of channel, spatial, and pixel attention. Removing spatial attention causes the largest reduction in SSIM, indicating that spatial attention is important for locating and preserving salient anatomical structures. Removing channel attention results in an evident decrease in MI and CC, suggesting that channel-wise recalibration facilitates the selection of complementary modality features. Although removing pixel attention causes a relatively smaller degradation, all metrics still decline consistently, confirming its contribution to fine-grained edge and texture enhancement.

#### 4.5.2. Component-Wise Ablation of the PGA Block

We further evaluated the individual contributions of the learned MRI-conditioned prior, MRI-derived edge information, and adaptive gating mechanism within the PGA Block. Five configurations were compared: fixed average fusion, edge guidance only, learned prior only, combined prior and edge guidance with fixed coefficients, and the complete adaptive PGA Block.

As reported in Table 8, fixed average fusion produces the lowest overall performance, indicating that static equal weighting cannot adequately accommodate spatial variations in multimodal information. The edge-guided variant achieves relatively better structural metrics, particularly SSIM and PSNR, confirming the role of edge information in preserving anatomical boundaries. In comparison, the learned-prior-only variant achieves higher MI and EN, suggesting that the learned spatial weighting map facilitates the preservation of complementary functional information.

Combining the learned prior with edge guidance improves all evaluated metrics, demonstrating that the two components provide complementary information. The complete PGA Block with learnable gating parameters achieves the best overall performance, further confirming that adaptive weighting is important for balancing anatomical structure preservation and functional information retention.

### 4.6. Noise Experiment

**Noise Settings:** To ensure the reproducibility of the robustness evaluation, all source images were first normalized to the range of $\left[01\right]$. In both noise experiments, noise was added only to the luminance channel of the PET images, while the corresponding MRI images remained noise-free. This setting was adopted to evaluate whether the fusion methods could suppress interference in the functional modality while preserving the reliable anatomical information provided by MRI. The original chrominance channels of the PET images were retained without modification.

For the Gaussian-noise experiment, additive Gaussian noise with a mean of 0 and a standard deviation of 0.05 was used, corresponding to a variance of 0.0025. For the Poisson-noise experiment, the noise intensity was controlled using a peak value of 30. After noise addition, all pixel intensities were clipped to the valid range of $\left[01\right]$.

Gaussian and Poisson noise were evaluated separately and were not applied simultaneously. For each test image pair and each noise type, five independent random noise realizations were generated. All comparison methods were evaluated using the same noisy inputs, and the quantitative results reported in Table 9 and Table 10 were averaged over all test image pairs and the five random noise realizations.

**Figure 8:**
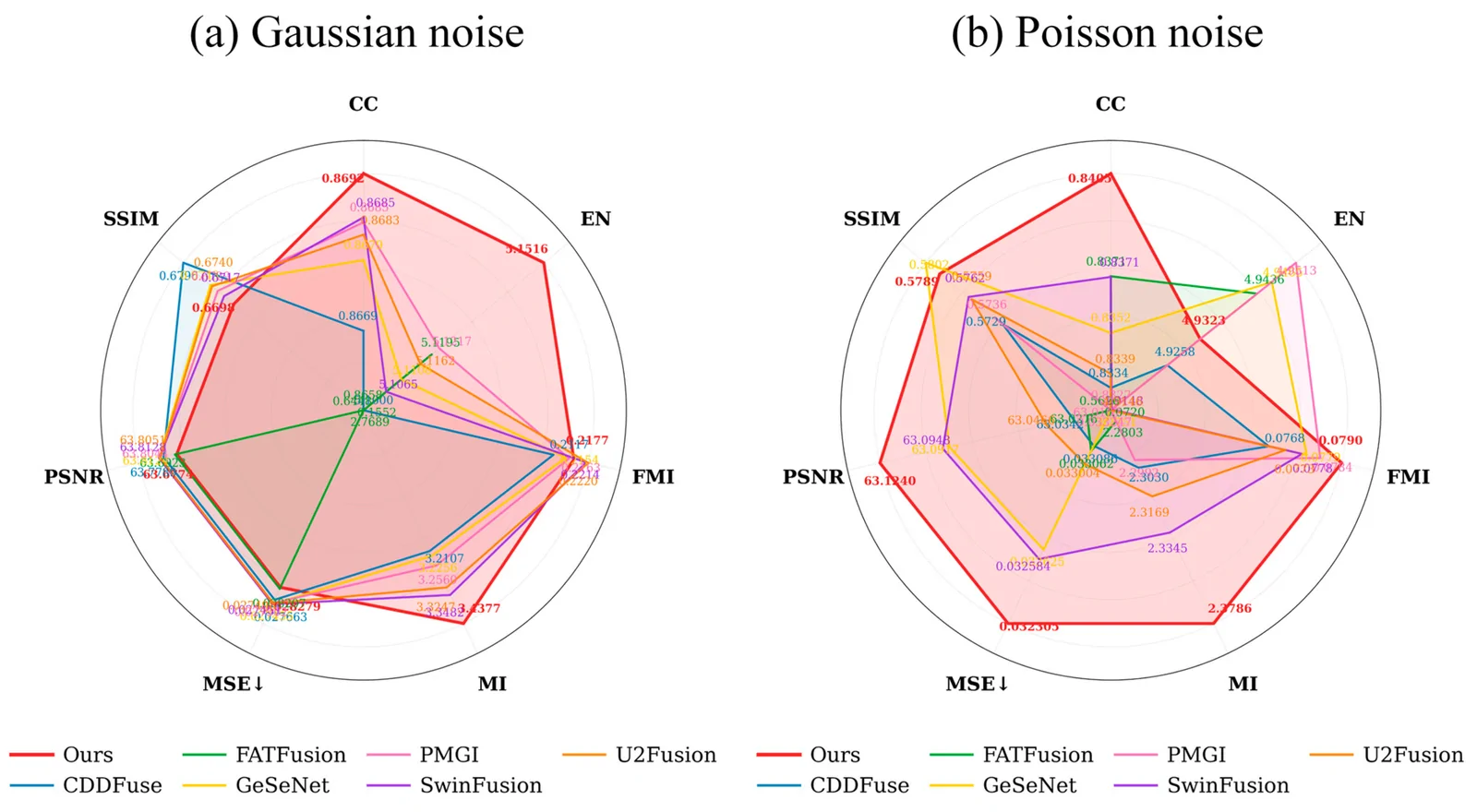
Robustness comparison under noise interference. (**a**) Results under Gaussian noise; (**b**) results under Poisson noise.

Experimental results on the MRI-PET dataset under noisy conditions (Figure 8, Table 9 and Table 10) demonstrate that the proposed method exhibits robust resilience against interference. Quantitative metrics show that our method outperforms baseline algorithms across the majority of key evaluation criteria. Under Gaussian noise, our method achieves top-tier performance in MI (3.4377) and FMI (0.2177). The Multi-Scale Encoder extracts features through parallel branches with different receptive fields. This improves the model’s ability to preserve complementary multimodal information under noisy conditions. The low MSE of 0.0275 also indicates effective suppression of fusion errors. This benefit is supported by the DEA Block, whose channel, spatial, and pixel attention mechanisms reduce high-frequency noise while preserving fine textures. Under Poisson noise scenarios, the model maintains a high information entropy EN (4.9249) while realizing a significant improvement in CC (0.8339). This outstanding performance profoundly validates the efficacy of the PGA Block, which employs anatomical structure priors to constrain the injection of functional information, adaptively filtering random noise impulses. Consequently, it not only preserves rich image information but also markedly enhances the structural consistency and correlation of the fusion results.

### 4.7. Modality Degradation Experiment

In scenarios of PET and MRI degradation, our method maintained significant advantages by adaptively adjusting fusion weights to suppress blocky artifacts and structural blurring. Visual analysis confirmed that TAPGFusion synergistically presents anatomical and functional information, delivering distinctly defined boundaries devoid of artifacts.

**Figure 9:**
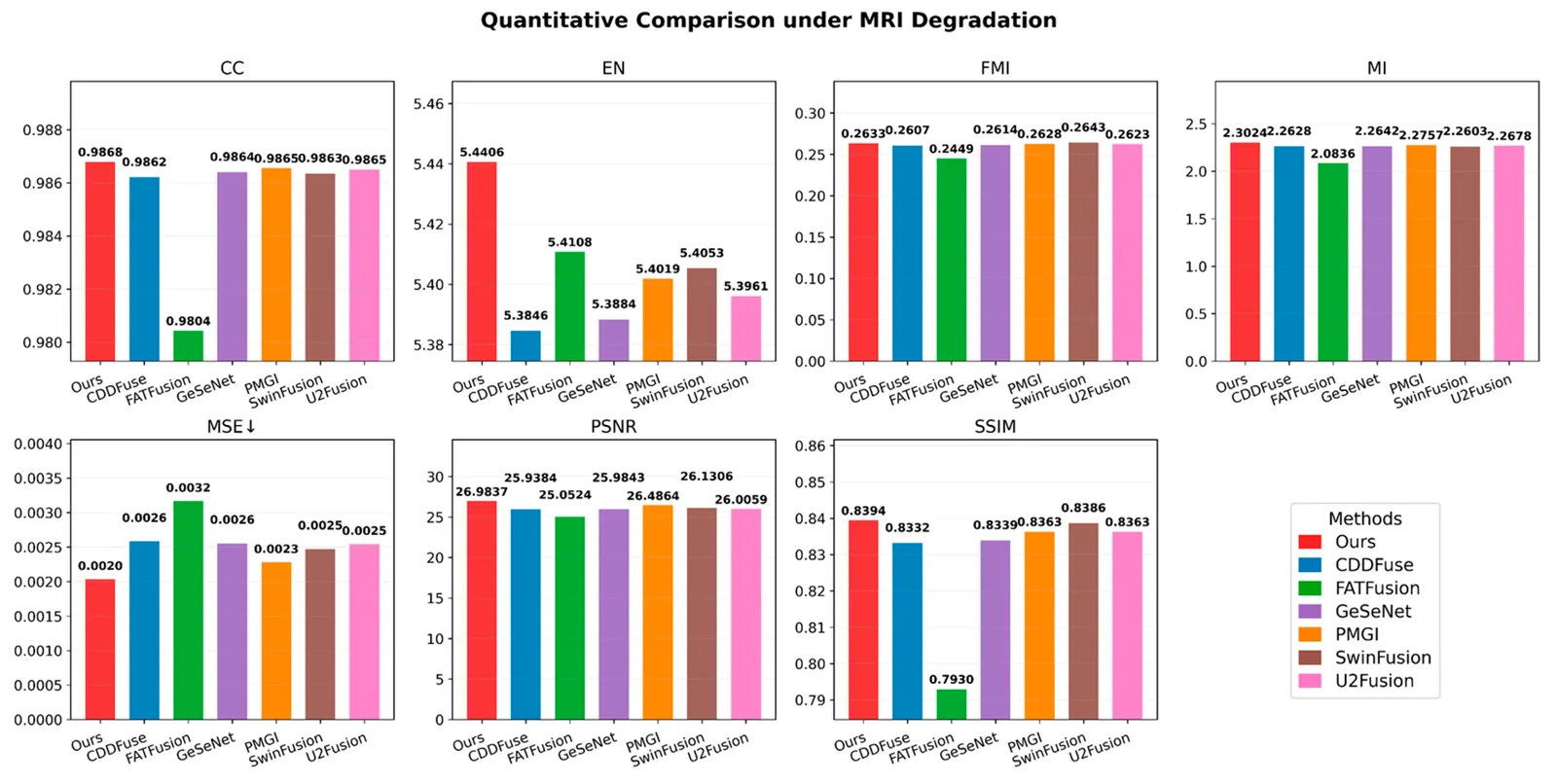
The visual comparison results in a modality degradation experiment for MRI.

In the PET modality degradation experiments conducted on the MRI-PET dataset, quantitative results (Figure 9) demonstrate that the proposed method achieves significant advantages across multiple critical metrics. The CC reaches 0.9989 and MSE drops to 0.0002, alongside an MI of 3.5765 and an EN of 5.1584. This indicates that the model effectively suppresses block artifacts and detail loss induced by degraded PET inputs, achieving high-precision structural reconstruction. The PGA Block improves robustness by adapting the fusion weights to differences in modality quality. When the PET image is degraded, the gating mechanism reduces the influence of unreliable PET features and relies more on the anatomical information from MRI. This helps maintain a high CC and a low MSE. Furthermore, it provides a stable feature foundation for cross-modal interaction, supporting elevated levels of information entropy and mutual information. The DEA Block further mitigates the information deficit caused by PET degradation by strengthening the transmission of MRI edge features, ultimately delivering high-quality fusion outputs even in modality-deficient scenarios.

**Figure 10:**
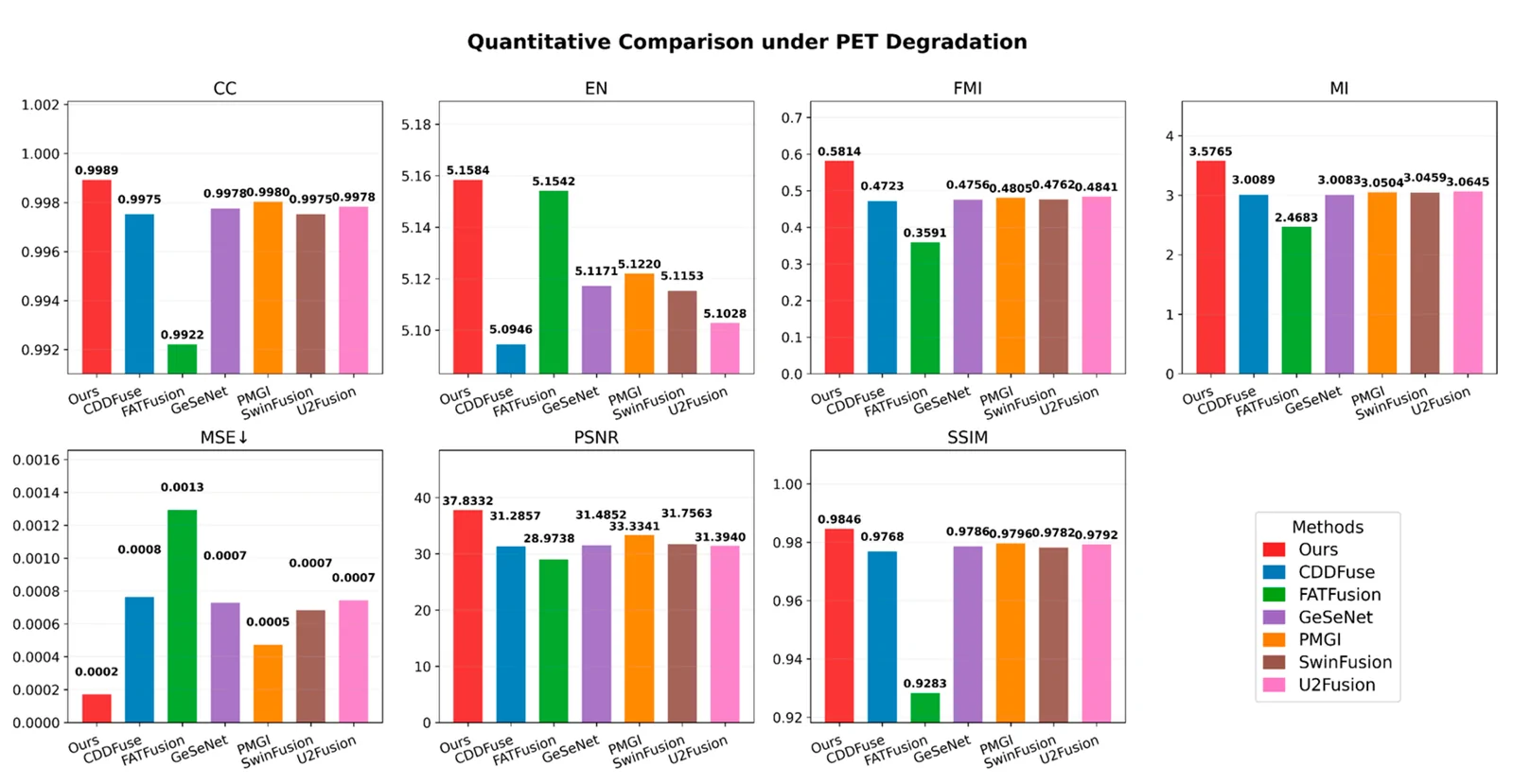
The visual comparison results in a modality degradation experiment for PET.

In the MRI modality degradation experiments (Figure 10), quantitative outcomes show that the model achieves a CC of 0.9865, FMI of 0.2639, MI of 2.3024, PSNR of 26.9837, and SSIM of 0.8394, with the MSE reaching a low of 0.0020, significantly outperforming comparative methodologies. This demonstrates the model’s effectiveness in mitigating structural blurring and detail loss caused by MRI degradation, precisely preserving complementary multimodal information. The PGA Block senses the degradation in MRI quality and adaptively elevates the fusion weight of the functional PET modality, dodging structural interference from the compromised MRI to ensure high CC and low MSE. The Multi-Scale Encoder provides a stable base for cross-modal feature interactions, supporting high information entropy (EN = 5.4406) and mutual information. Meanwhile, the DEA Block bolsters the transmission of PET functional features alongside residual MRI edge features, bridging the anatomical information gap caused by MRI degradation and executing high-quality fusion despite missing modality data.

### 4.8. Visualization Analysis of Intermediate Features

**Figure 11:**
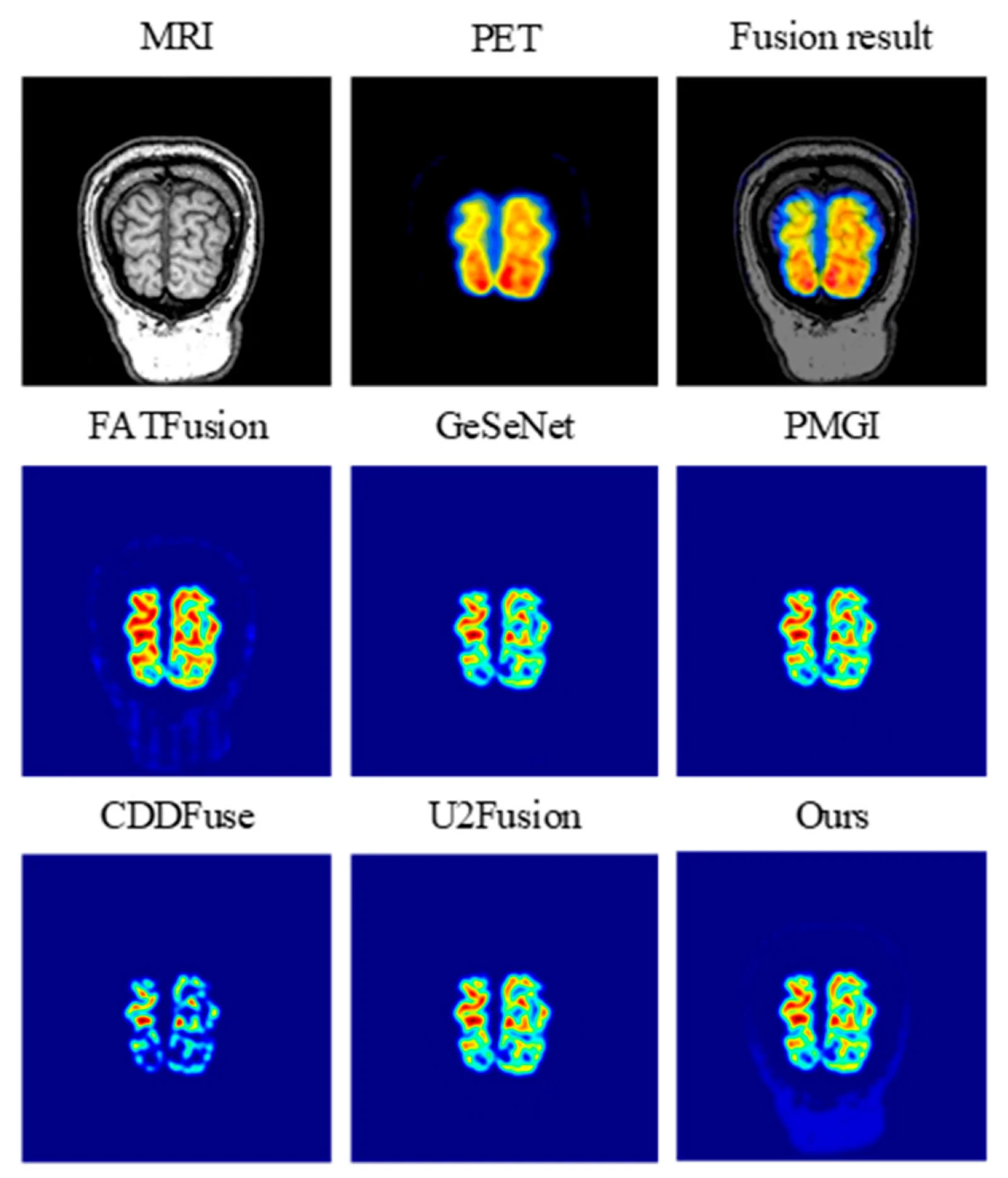
Visualization analysis of intermediate features.

Intermediate Feature Visualization Analysis: Based on the fusion visualization results from the MRI-PET dataset (Figure 11), the proposed method displays clear superiority in both visual fidelity and information completeness. Compared to baseline methods, the fusion output of our approach not only fully retains the crisp anatomical structural contours of the MRI but also precisely restores the functional metabolic hotspots of the PET, achieving highly synergistic presentation of both anatomical and functional information. In contrast, FATFusion, GeSeNet, PMGI, CDDFuse, and U2Fusion all exhibit varying degrees of information loss or structural blurring. Some comparative methods even demonstrate fragmented edges in PET functional regions and missing anatomical outlines in MRI regions. Conversely, the output of the proposed method features clear, well-stratified boundaries for both brain anatomical structures and metabolic zones, devoid of artifact interference or detail omission, qualitatively illustrating the effectiveness of the proposed framework in preserving complementary multimodal information.

## 5. Conclusions

To address the fundamental challenge in multimodal medical image fusion—the inherent difficulty of simultaneously preserving anatomical structural integrity and functional information validity—this study introduces the TAPGFusion framework. Our architecture leverages a multi-scale parallel encoder to achieve the complementary extraction of fine-grained details and macro-anatomical structures. The DEA Block suppresses redundant noise and enhances important tissue features through channel, spatial, and pixel attention. The PGA Block then assigns modality weights according to regional anatomical and functional characteristics. Ablation experiments on the CT–MRI, PET–MRI, and SPECT–MRI datasets show that removing any core module reduces overall performance. The complete model achieves the best or near-best results for most metrics, including CC, EN, FMI, MI, PSNR, and SSIM, while maintaining a competitive MSE. These results confirm the complementary contributions of the proposed modules. Comparative evaluations further reveal that the proposed model effectively enhances the structural clarity and functional discriminability of the fused images while maintaining a lightweight computational footprint, thereby offering a highly efficient solution for multimodal medical image fusion. Future research will focus on optimizing cross-modal feature alignment and enhancing the model’s generalization capabilities in few-shot scenarios, ultimately broadening the clinical applicability of the framework.

## Figures and Tables

**Table 1:** Average values of seven metrics on 24 CT-MRI test image pairs. The best results are in bold, the downward arrow (↓) indicates that a lower value is better.

Methods	CC	EN	FMI	MI	MSE ↓	PSNR	SSIM
CDDFuse	0.7984	5.5460	0.4863	2.6226	0.0334	62.9760	0.6221
FATFusion	0.8235	**5.9146**	0.3591	2.3495	0.0292	63.5571	0.6978
Gese Net	0.8256	4.8166	0.6123	2.7286	0.0271	63.8714	0.7795
PMGI	0.8294	4.8281	0.6001	**2.7338**	0.0267	63.9416	0.7320
IFCNN	0.8187	5.7821	0.3826	2.4173	0.0297	63.4826	0.6849
SwinFuse	0.8313	4.4881	0.6528	2.0018	0.0274	63.8311	0.6624
U2Fusion	0.8201	4.5985	**0.6599**	2.5927	0.0325	63.0574	0.7877
**Ours**	**0.8333**	4.8401	0.6142	2.6878	**0.0265**	**63.9868**	**0.7884**

**Table 2:** Average values of seven metrics on 24 PET-MRI test image pairs. The best results are in bold, the downward arrow (↓) indicates that a lower value is better.

Methods	CC	EN	FMI	MI	MSE ↓	PSNR	SSIM
CDDFuse	0.8503	4.9299	0.5619	3.2433	0.0315	63.2046	0.7336
FATFusion	0.8577	5.0904	0.5524	2.6913	0.0291	63.5558	0.7817
GeSeNet	0.8679	5.1181	0.5477	3.0373	0.0281	63.7028	0.7698
PMGI	0.7557	2.7822	**0.6442**	2.9077	0.0673	59.8850	0.7189
IFCNN	0.8593	5.0476	**0.5571**	2.8438	0.0296	63.4917	0.7654
SwinFuse	0.8664	5.0315	0.5488	3.3808	0.0283	63.6732	0.7691
U2Fusion	0.8665	5.0719	0.5533	2.9823	0.0284	63.6561	0.7398
**Ours**	**0.8691**	**5.1583**	0.5512	**3.4233**	**0.0280**	**63.7154**	**0.7886**

**Table 3:** Average values of seven metrics on 24 SPECT-MRI test image pairs. The best results are in bold, the downward arrow (↓) indicates that a lower value is better.

Methods	CC	EN	FMI	MI	MSE ↓	PSNR	SSIM
CDDFuse	0.8850	3.9986	0.6341	2.7033	0.0122	67.4971	0.8466
FATFusion	0.8982	4.1217	**0.6401**	2.5215	0.0096	67.4719	0.8520
GeSeNet	0.9028	4.1070	0.6358	2.6673	**0.0094**	67.6110	0.8337
PMGI	0.9028	4.0295	0.6315	2.6801	0.0114	67.1154	0.8152
IFCNN	0.8995	4.0964	0.6369	2.5837	0.0101	67.3968	0.8436
SwinFuse	0.9032	4.1521	0.6287	2.7894	0.0106	67.0177	0.8501
U2Fusion	0.8969	4.0843	0.6331	2.6150	0.0112	67.7074	0.8458
**Ours**	**0.9073**	**4.2259**	0.6312	**2.8128**	0.0115	**67.7177**	**0.8692**

**Table 4:** Ablation study on the CT-MRI dataset. The upward arrow (↑) indicates that a higher value is better, while the downward arrow (↓) indicates that a lower value is better. The best results are in bold. The checkmark (✓) indicates that the corresponding module is included in the configuration.

Multi-Scale	DEA	PGA	CC ↑	EN ↑	FMI ↑	MI ↑	MSE ↓	PSNR ↑	SSIM ↑
			0.8269	4.7305	0.6299	2.6626	0.0266	64.0327	0.7774
✓			0.8290	4.7550	0.6352	2.6660	0.0267	63.9800	0.7800
	✓		0.8274	4.7420	0.6405	2.6450	0.0269	63.9100	0.7815
		✓	0.8282	4.7485	0.6360	2.6505	0.0268	63.9400	0.7835
✓	✓		0.8321	4.7703	0.6386	2.6707	0.0268	63.9371	0.7733
✓		✓	0.8280	4.7535	0.6410	2.5732	0.0275	63.8172	0.7882
	✓	✓	0.8195	4.5927	**0.6543**	2.5829	0.0272	63.8641	0.7698
✓	✓	✓	**0.8333**	**4.8401**	0.6142	**2.6878**	**0.0265**	63.9868	**0.7884**

**Table 5:** Ablation study on the PET-MRI dataset. The upward arrow (↑) indicates that a higher value is better, while the downward arrow (↓) indicates that a lower value is better. The best results are in bold. The checkmark (✓) indicates that the corresponding module is included in the configuration.

Multi-Scale	DEA	PGA	CC ↑	EN ↑	FMI ↑	MI ↑	MSE ↓	PSNR ↑	SSIM ↑
			0.8686	5.1441	0.5464	3.2225	0.0286	63.7065	0.7831
✓			0.8687	5.1512	0.5470	3.2600	0.0285	63.7200	0.7840
	✓		0.8683	5.1580	0.5488	3.2450	0.0284	63.7100	0.7790
		✓	0.8685	5.1535	0.5495	3.2850	0.0283	63.7300	0.7820
✓	✓		0.8674	**5.1804**	0.5440	3.2354	0.0288	63.5872	0.7266
✓		✓	0.8686	5.1694	0.5457	3.1705	0.0287	**63.7504**	0.7675
	✓	✓	0.8681	5.1658	0.5511	3.1073	**0.0280**	63.7006	0.7644
✓	✓	✓	**0.8691**	5.1583	**0.5512**	**3.4233**	**0.0280**	63.7154	**0.7886**

**Table 6:** Ablation study on the SPECT-MRI dataset. The upward arrow (↑) indicates that a higher value is better, while the downward arrow (↓) indicates that a lower value is better. The best results are in bold. The checkmark (✓) indicates that the corresponding module is included in the configuration.

Multi-Scale	DEA	PGA	CC ↑	EN ↑	FMI ↑	MI ↑	MSE ↓	PSNR ↑	SSIM ↑
			0.9072	4.2230	0.6300	2.7827	0.0115	67.7005	0.8603
✓			0.9071	4.2160	0.6305	2.7900	0.0114	67.7050	0.8620
	✓		0.9070	4.2200	0.6307	2.7750	0.0114	67.6900	0.8615
		✓	0.9072	4.2215	0.6306	2.7955	0.0113	67.7100	0.8630
✓	✓		0.9067	4.1931	0.6213	2.7617	0.0116	67.3404	0.8655
✓		✓	0.9072	4.2110	0.6311	2.7382	**0.0110**	67.6162	0.8637
	✓	✓	0.9068	4.2066	0.6309	2.7311	0.0112	67.6688	0.8659
✓	✓	✓	**0.9073**	**4.2259**	**0.6312**	**2.8128**	0.0115	**67.7177**	**0.8692**

**Table 7:** Component-wise ablation study of the DEA Block on the PET–MRI dataset. The upward arrow (↑) indicates that a higher value is better, while the downward arrow (↓) indicates that a lower value is better. The best results are in bold.

Configuration	SSIM ↑	PSNR ↑	MSE ↓	MI ↑	FMI ↑	EN ↑	CC ↑
Residual convolution only	0.7594	63.4387	0.0299	3.1182	0.5428	5.1037	0.8630
DEA without channel attention	0.7758	63.5884	0.0291	3.2746	0.5463	5.1295	0.8665
DEA without spatial attention	0.7721	63.5527	0.0293	3.2479	0.5471	5.1231	0.8659
DEA without pixel attention	0.7810	63.6426	0.0286	3.3318	0.5490	5.1444	0.8677
Full DEA Block	**0.7886**	**63.7154**	**0.0280**	**3.4233**	**0.5512**	**5.1583**	**0.8691**

**Table 8:** Component-wise ablation study of the PGA Block on the PET–MRI dataset. The upward arrow (↑) indicates that a higher value is better, while the downward arrow (↓) indicates that a lower value is better. The best results are in bold.

Configuration	SSIM ↑	PSNR ↑	MSE ↓	MI ↑	FMI ↑	EN ↑	CC ↑
Fixed average fusion	0.7618	63.4526	0.0298	3.0915	0.5441	5.1168	0.8638
Edge guidance only	0.7807	63.6381	0.0288	3.1854	0.5467	5.1262	0.8669
Learned prior only	0.7752	63.5914	0.0290	3.3147	0.5482	5.1459	0.8663
Prior + edge with fixed coefficients	0.7845	63.6738	0.0284	3.3679	0.5496	5.1510	0.8681
Full adaptive PGA Block	**0.7886**	**63.7154**	**0.0280**	**3.4233**	**0.5512**	**5.1583**	**0.8691**

**Table 9:** Quantitative comparison of different fusion methods under Gaussian noise interference. The best results are in bold, the downward arrow (↓) indicates that a lower value is better.

Methods	CC	EN	FMI	MI	MSE ↓	PSNR	SSIM
CDDFuse	0.8689	5.1000	0.2117	3.2107	**0.0283**	63.7789	**0.6796**
FATFusion	0.8658	5.1195	0.1552	2.7689	0.0277	63.6923	0.6448
GeSeNet	0.8679	5.1108	0.2174	3.2256	0.0282	**63.8124**	0.6743
PMGI	0.8685	5.1217	0.2163	3.2560	0.0279	63.8044	0.6729
SwinFusion	0.8685	5.1065	0.2214	3.3482	0.0273	63.8128	0.6717
U2Fusion	0.8683	5.1162	**0.2220**	3.3247	0.0274	63.8051	0.6740
**Ours**	**0.8692**	**5.1516**	0.2177	**3.4377**	0.0275	63.6774	0.6698

**Table 10:** Quantitative comparison of different fusion methods under Poisson noise interference. The best results are in bold, the downward arrow (↓) indicates that a lower value is better.

Methods	CC	EN	FMI	MI	MSE ↓	PSNR	SSIM
CDDFuse	0.8268	4.8946	0.0760	2.2884	0.0338	62.9833	0.5648
FATFusion	0.8318	4.9624	0.0710	2.2246	0.0334	62.9883	**0.5742**
GeSeNet	0.8333	4.9494	0.0773	2.2744	0.0330	63.0406	0.5730
PMGI	0.8300	4.9491	0.0770	2.2932	0.0337	62.9559	0.5675
SwinFusion	0.8329	4.8982	0.0768	2.3080	0.0332	63.0180	0.5675
U2Fusion	0.8307	4.8931	0.0764	2.3072	0.0335	62.9824	0.5667
**Ours**	**0.8339**	**4.9249**	**0.0779**	**2.3221**	**0.0324**	**63.1109**	0.5715

## Data Availability

The original multimodal medical image datasets (including CT-MRI, 573 PET-MRI, and SPECT-MRI) analyzed in this study are publicly available. This data can be accessed from the Harvard Whole Brain Atlas at http://www.med.harvard.edu/AANLIB/home.html (accessed on 10 December 2025).
